# DRER: Deep Learning–Based Driver’s Real Emotion Recognizer

**DOI:** 10.3390/s21062166

**Published:** 2021-03-19

**Authors:** Geesung Oh, Junghwan Ryu, Euiseok Jeong, Ji Hyun Yang, Sungwook Hwang, Sangho Lee, Sejoon Lim

**Affiliations:** 1 Graduate School of Automotive Engineering, Kookmin University, 77, Jeongneung-ro, Seongbuk-gu, Seoul 02707, Korea; gsethan17@kookmin.ac.kr (G.O.); jhryu1003@kookmin.ac.kr (J.R.); euiseok_jeong@kookmin.ac.kr (E.J.); 2 Department of Automobile and IT Convergence, Kookmin University, 77, Jeongneung-ro, Seongbuk-gu, Seoul 02707, Korea; yangjh@kookmin.ac.kr; 3 Chassis System Control Research Lab, Hyundai Motor Group, Hwaseong 18280, Korea; gazz@hyundai.com (S.H.); imprince@hyundai.com (S.L.)

**Keywords:** human–machine interface, emotion recognition, real emotion, driver’s emotional state, deep learning, sensor fusion

## Abstract

In intelligent vehicles, it is essential to monitor the driver’s condition; however, recognizing the driver’s emotional state is one of the most challenging and important tasks. Most previous studies focused on facial expression recognition to monitor the driver’s emotional state. However, while driving, many factors are preventing the drivers from revealing the emotions on their faces. To address this problem, we propose a deep learning-based driver’s real emotion recognizer (DRER), which is a deep learning-based algorithm to recognize the drivers’ real emotions that cannot be completely identified based on their facial expressions. The proposed algorithm comprises of two models: (i) facial expression recognition model, which refers to the state-of-the-art convolutional neural network structure; and (ii) sensor fusion emotion recognition model, which fuses the recognized state of facial expressions with electrodermal activity, a bio-physiological signal representing electrical characteristics of the skin, in recognizing even the driver’s real emotional state. Hence, we categorized the driver’s emotion and conducted human-in-the-loop experiments to acquire the data. Experimental results show that the proposed fusing approach achieves 114% increase in accuracy compared to using only the facial expressions and 146% increase in accuracy compare to using only the electrodermal activity. In conclusion, our proposed method achieves 86.8% recognition accuracy in recognizing the driver’s induced emotion while driving situation.

## 1. Introduction

Drivers’ emotional state affects their ability to drive [1,2]. As vehicles become more intelligent, it becomes increasingly important to recognize the driver’s emotions. Accurately detecting the driver’s emotional state allows the vehicle to respond more quickly to the driver’s emotional needs; it can provide adequate infotainment support and adjusts vehicle dynamics for a safer and more comfortable ride. In intelligent vehicles, recognizing the driver’s emotion is emphasized because vehicles can select the options according to driver’s emotional state (e.g., driving mode, song to change the atmosphere and driving by themselves).

In the human–machine interface, facial expressions are considered important because they are useful for revealing emotions between people. These methods based on facial expressions have been established as a research field called facial expression recognition (FER). With the great development of deep learning-based image recognition technology, deep learning is more utilized for FER [3,4,5,6,7,8]. However, facial expressions cannot always reveal human’s real emotions due to various factors. Particularly, this characteristic is even more significant in case of drivers. For instance, when a driver frowns while driving, it may be tempting to assume that the driver is currently in an unpleasant state if the judgment is made purely based on the driver’s facial expressions. However, if it is simply the reaction of the driver’s facial muscles to the stimulus of sunlight, then the driver should not be judged to be in an unpleasant state. Therefore, it is not always the driver’s emotions that appear on their facial expressions. Consequently, we aim to recognize the driver’s real emotion even in situations in which the real emotion is not fully revealed via facial expressions while driving. There is a similar research field named micro facial expressions, in which micro changes in expressions within a very short duration are studied. Such micro changes normally occur when the real emotions are concealed deliberately or unconsciously. Some research focusing on facial micro expressions has realized promising methods for detecting concealed emotions [9,10,11,12]. A micro expression can be a clue to the driver’s real emotions, but the lack of samples per category and the imbalanced distribution of samples are the primary obstacles associated with its usage in deep learning-based algorithms [13]. Ultimately, the driver’s real emotion that we aim for is not concealed emotion but emotion that is not fully revealed. Furthermore, most research uses bio-physiology signals for recognizing human emotions [14,15,16,17,18,19,20,21,22,23]. The most commonly used bio-physiology signals are electroencephalogram (EEG), electrocardiogram (ECG), photoplethysmography (PPG) and electrodermal activity (EDA). Moreover, some studies using both facial expressions and bio-physiology signals achieved high accuracy in the case of emotion recognition [24,25] and performed well in recognizing various emotion classes [26,27]. All these studies are based on deep learning algorithms. Based on the above trends, we propose a deep learning-based driver’s real emotion recognizer (DRER) to recognize the driver’s real emotional state while driving based on the sensor fusion of the driver’s FER and bio-physiology data. The proposed method is divided into two steps.

The first step is the FER—recognizing the driver’s facial expressions while driving. We propose a FER model constructed with reference to several state-of-the-art convolutional neural networks (CNNs), such as VGGNet [28], ResNet [29], ResNeXt [30] and SENet [31]. The proposed FER model is an end-to-end architecture; thus, the model receives a whole image of the driver’s face and outputs its recognized facial expression state. The FER model recognizes the driver’s facial expression state using continuous representations, valence and arousal, which are the most popular emotional continuous representations proposed by Russell [32]. Among several databases [33,34,35,36,37], we trained the FER model using the AffectNet [33], which has more than 1M facial images and annotated valence and arousal. The model adding SENet to ResNeXt networks obtains the same level of accuracy as the baseline proposed by Mollahosseini et al. [33].

The second step is the sensor fusion emotion recognition (SFER)—recognizing the driver’s real emotions by fusing the recognized state of facial expressions with the driver’s bio-physiology signals. On the basis of the deep neural network (DNN), we propose the SFER model to receive the driver’s recognized facial expression state (represented by the valence and arousal) and EDA signals of the driver and output the driver’s recognized real emotional state. The driver’s recognized real emotional state is represented among the several discrete categories. To avoid confusion among the classified emotions, we categorize the emotions according to the driver’s real emotion while driving: neutral, happy, excited, fearful, angry, depressed, bored and relieved. Motivated by the training and experimental SFER model, we need a dataset that contains the driver’s real emotions (represented by the aforementioned emotion categories). Hence, we conducted a human-in-the-loop simulation to obtain the dataset. Thirteen volunteers participated, in which they drove a full-scale driving simulator with each emotion induced. We measured the driver’s facial image and EDA during the simulation and split the measured data into training, validation and test sets through average filtering. We trained the SFER model using the training and validation set from the human-in-the-loop simulation.

In this study, we obtained remarkably consistent results with respect to the sensor fusion of the driver’s facial expressions and EDA data. When the driver’s emotions are recognized by EDA data alone, it has an accuracy of 33.1–35.8%, whereas, when it is recognized only by the results of FER, it has an accuracy of 37.6–41.1%. When the driver’s emotions are recognized by combining the two aforementioned parameters, the accuracy is 65.8–88.0%. Through an advancement of the algorithm, the proposed DRER shows the highest accuracy of 88.8%.

Our main contributions are as follows. First, a deep learning-based intermediate sensor fusion algorithm is proposed. There are several sensor fusion strategies: early fusion, late fusion and intermediate fusion. Early fusion, also known as data-level fusion, is a traditional fusion strategy that fuses data before they are analyzed. Late fusion, also known as decision-level fusion, is a fusion strategy that fuses decisions made using each individual sensor data, and it is simpler than early fusion when the sampling time, unit and dimensionality of data are different. Intermediate fusion, also known as feature-level fusion, is the most flexible strategy that fuses extracted higher level features and allows them to be the fusion stages of model training. For our model, the FER model extracts higher level features from facial images. Then, the SFER model fuses the extracted features and EDA to recognize emotional states. This is the reason that our model is referred to as an intermediate sensor fusion algorithm. The proposed algorithm recognizes the driver’s real emotion state among the newly defined eight emotional categories suitable for a driver by only fusing two sensors, i.e., the facial camera and EDA sensor, which can be easily collected from a vehicle. Second, the experiment results show a higher accuracy for recognition of the driver’s real emotion when fusing the facial camera and EDA sensor data than when using each datum individually. The proposed algorithm was evaluated with the data collected through a full-scale driving simulator that is similar to an actual vehicle environment. As a result, a recognition accuracy of 86.8% was achieved, which is greater than those obtained using only the facial camera and an EDA sensor by 114% and 146%, respectively. Finally, when compared with other state-of-the-art algorithms, the prediction accuracy was the highest despite having the largest number of classification classes.

The rest of this paper is organized as follows. Section 2 introduces the emotion recognition’s related work. Section 3 discusses the proposed DRER for recognizing the driver’s real emotion with the driver’s facial expressions and EDA data while driving. Section 4 provides the details of the proposed model. Section 5 presents the extensive experiments and human-in-the-loop simulation. Section 6 compares and analyzes the experiment results. Section 7 concludes this work and describes further work.

## 2. Related Work

This section describes emotion recognition’s related work. We introduce the general flow of FER, the emotion recognition using the bio-physiological signals and SFER. To secure the data for emotion recognition, we also introduce the database and the human-in-the-loop simulation.

### 2.1. Facial Expression Recognition

Most previous studies produced remarkable results using CNN and adding other networks, such as support vector machine (SVM) and recurrent neural network (RNN), with preprocessed data. The 2014 Emotion Recognition in the Wild Challenge (EmotiW) winners compared the results using several deep learning techniques, such as CNN, deep belief net, relational autoencoder and shallow network. They added that the best performing model was a model that could extract the probability of an emotional class with CNN to train the SVM’s hyperparameters [3]. However, FER is different because it depends on the emotion definition. There are two ways to define emotion: discrete and continuous. Discrete emotions are used to classify categorical emotions, e.g., anger, disgust, happy and neutral. For this case, 2016 EmotiW winners preprocessed the Acted Facial Expressions in the Wild (AFEW) dataset using a face detector and face similarity transform, and the CNN filter filtered out the faceless frame. The preprocessed data were put into the CNN (VGG16) and 3D convolution model. CNN extracts features and uses RNN/LSTM encoder to map inputs into fixed-length vectors to encode motion, whereas 3D convolution models use video shape and motion [4]. Similarly, previous studies about FER used two parallel CNNs and merged the extracted features before recognition. Jeong et al. [38] simultaneously used 3D and 2D convolution models with the different preprocessed data. The resultant features of each model are merged to predict through softmax. Riaz et al. [39] proposed an algorithm with two network routes separated by pooling is present or not. During intermediate feature extraction, each route’s results are merged before the next layer. Specifically for drivers, Gao et al. [5] made a model to determine whether the driver is in a stressful situation or not. They defined anger and disgust as a stressful situation. They trained the SVM with PCA results applied features to the additional released data after landmark extraction using the SIFT descriptor. They divided the indoor conditions (expression from the driver’s front) and the vehicle conditions (expression from the instrument panel) to collect experimental data for evaluation. Similarly, studies for driver usually trained their model with released data and evaluated with the experimental data, and they defined emotion themselves following the goal of their studies.

The continuous definition is another way of defining emotions. Most previous studies about continuous emotion used Russel’s V-A (valence–arousal) model. Valence is the degree of emotional appeal, which can be negative or positive. Arousal refers to a condition in which various nerves are physiologically active. The higher is the emotion intensity, the higher is the arousal level. The continuous definition has been used for V-A regression because V-A ranges from −1 to 1. One of the studies about V-A regression presented the FATAUVA-net model, which comprises a core, attribute, action unit (AU) and V-A layer, based on CNN (MCNN). In core and attribute layers, the face is detected through MTCNN and facial part’s localize area (such as face and eye) to train CNN with the CelebA dataset. The AU layer learns about AU through the facial part extracted from the attribute layer with the Affect-in-the-wild (AFF-wild) dataset and finally estimates V-A in the V-A layer [6]. Some studies predicted both V-A regression and categorical classification of emotions. Kollias and Zafeiriou [7] created a dataset by annotating AUs and labeling AFF-wild dataset to the most similar seven basic emotions. They performed semi-supervised guidance through GAN after preprocessing with the FFLD2 face detector. They extracted features via CNN (AFF-WildNet) and used them as input to RNN (VGGFACE-GRU) to derive nine outputs. Two of them are the V-A estimation result, while the others predict the seven basic emotions through softmax. Some studies used V-A as an intermediate extract to classify categorical emotions because V-A is not intuitive in meaning. Theagarajan et al. [8] developed an automated system to analyze facial expressions using the car driver’s V-A. They localized the diver’s facial videos from the AFEW-VA and Motor Trends Magazine datasets by the pre-trained YOLO V2 and then used them to extract features through CNN (ResNet). Extracted features are inserted as input vectors of the RNN (LSTM) and estimating V-A to predict the six emotions through SVM.

### 2.2. Bio-Physiological Signals

In emotion recognition studies, bio-physiological signals are frequently used as the input data. Sensors that can acquire bio-physiological signals are diverse, and each signal from a sensor has its own characteristics. A thorough review of sensors and methods for human emotion recognition was provided by Dzedzickis et al. [17]. According to their paper, EEG, which is a basic technique, is usually evaluated and analyzed in five frequency ranges to estimate the average level of valence and arousal or to detect efficiency. Recently, researchers have focused on developing new methods for information extraction from EEG with deep learning techniques. In addition, researchers focus on defining and evaluation QRS amplitudes and their duration. Emotion can be evaluated by defining P or R peaks and other parameters from QRS. EDA allows not only emotion recognition, but also an automatic detection decision-making process because EDA contains useful information concerning EDA signal’s amplitude and frequency. By implementing machine learning algorithms, it is possible to increase the precision of emotion recognition and recognize specific emotions relating to the level of arousal. PPG signal is filtered using high-pass filters before defining peaks and forming heart rate variability (HRV). Sensors are usually combined to complement each other’s drawbacks. Deng et al. [14] showed that performance tends to improve as data types increase, regardless of the recognition methods.

There are many previous studies in the field of emotion recognition using bio-physiological signals, which involved the above sensors. Raheel et al. [18] tried to recognize emotion while watching tactile enhanced multimedia. Four different video clips were selected and subjects had to rate the clips on a nine-point SAM scale about the videos. The physiological signals (EEG, EDA and PPG) were recorded while the subjects watched the video clips, and various features were extracted from these signals. A K-nearest neighbor classifier was applied to the extracted features to classify emotions (happy, relaxed, angry and sad). The result shows that the classification accuracy of PPG-based features was the highest (78.77%), and they could obtain higher accuracy (79.76%) with the fusion of EEG, EDA and PPG.

Recently, with advances in the measurement technology of EEG sensor and deep learning, emotion recognition research using EEG has been actively conducted because it involved brain’s response to various stimuli. Owing to the ability to evaluate the average level of valence and arousal, Liu et al. [19] proposed a subject-independent emotion recognition algorithm based on the dynamic empirical convolutional neural network (DECNN) using the EEG signals. They filtered the SJTU Emotion EEG dataset (SEED) with an empirical mode decomposition (EMD) algorithm to preserve the instantaneous characteristics, which is the frequency band of the EEG signal. To extract the features of the EEG signals, they used the dynamic differential entropy (DDE) algorithm. As a result, they could represent the time–frequency feature of the emotional state. Moreover, the time–frequency feature can be represented using a two-dimensional matrix, and the local correlation features can be indicated as images; they constructed a CNN model to identify the positive and negative emotions. They achieved 97.56% accuracy with these methods.

In another paper on EEG, Chao et al. [20] proposed a deep belief-conditional random field (DBN-CRF) framework with glia chains (DBN-GC) and conditional random field to capture the long-term dependencies, contextual information and correlation information between different channels of EEG signals. They split the multi-channel EEG signals of the DEAP, AMIGOS and SEED datasets into several segments by a specific time window and extracted a raw feature vector from the signals. Each raw feature vector was fed into the parallel DBN-GC to extract high-level representations. Then, the high-level feature sequence containing inter-channel correlation information of EEG signal was fed into the CRF. CRF generated the predicted emotion label sequence, and the decision merge layer based on a K-nearest neighbor algorithm can be used to determine the emotional state. They achieved a mean accuracy of 76.58% in terms of leave-one-out cross-validation.

However, EEG needs special equipment to obtain the signals; therefore, some studies have considered using other sensors, which are relatively less demanding, despite the advantages of EEG. Ooi et al. [15] processed the driver’s emotion using EDA in virtual driving simulations (three scenarios: neutral, stress and anger). These data were classified as two-class classification with SVM. They achieved an accuracy of 85% for neutral–stress and neutral–anger emotions, whereas stress–anger emotion was approximately 70%. It means that large sections’ emotions can be classified, but similar sections are difficult to classify. In another paper using EDA, Machot et al. [22] tried to recognize subject-independent human emotion using EDA as an input feature. They proposed a CNN model to predict four emotional states (high valence/high arousal (HVHA), high valence/low arousal (HVLA), low valence/low arousal (LVLA) and low valence/high arousal (LVHA)). They performed evaluation using MANHOB and DEAP datasets and obtained an accuracy of 81%. In addition, some researchers have used ECG with EDA [23]. They proposed a model based on CNN and LSTM to recognize the valence and arousal states. They obtained accuracy of 75% and 76% for valence and arousal, respectively.

Moreover, there is a study that used wearable sensors to make emotion recognition more practical and flexible in a wide field. Zheng et al. [21] constructed an emotion recognition approach based on a multimodal wearable biosensor network to facilitate emotion monitoring in daily life. The signals from the multimodal wearable biosensor were collected by measuring nodes and then transmitted to the sink node. They introduced a fuzzy rough nearest neighbors (FRNN) algorithm to classify different emotions considering the fuzzy thresholds of EEG concentration. Their method reduced the classification range of samples and disturbance of noisy samples, achieving 65.6% accuracy, which is high in wearable scenarios.

### 2.3. Sensor Fusion Emotion Recognition

To obtain bio-physiological signals, expensive equipment and a well-controlled environment are necessary. To overcome these drawbacks, many studies tried to recognize emotion using facial expression data and bio-physiological signals because it allows the implementation of a multimodal approach and the performance of non-contact measurements. It also produces quite reliable results with recent advances in computer vision systems, big data analysis and deep learning techniques. However, it still exhibits low accuracy with respect to the level of arousal. Therefore, some researchers have considered using both of facial expressions and bio-physiological signals [17]. Zhong et al. [16] tried to predict continuous emotions (valence and arousal) using facial expression data and bio-physiological signals. In their experiment, 20 video clips were shown to 27 participants to annotate their emotional state by choosing 10 emotional keywords (sadness, joy, happiness, disgust, neutral, amusement, anger, fear, surprise and anxiety). Simultaneously, they obtained the facial expression data recorded with six video cameras and the four bio-physiological signals (EDA, ECG, respiratory amplitude and skin temperature) were recorded. In FER, they adopted the AFFDEX SDK to fuse facial expression data and bio-physiological signals and proposed the temporal information preserving framework, which led to significant improved emotion recognition performance.

Because the usage of bio-physiological signals and facial expressions allows for the implementation of a multimodal approach, many studies have proposed multimodal emotion recognition. Kortelainen et al. [40] proposed multimodal automatic emotion recognition by combining the HRV parameters, respiration frequency and facial emotions. They showed 48 pictures with emotional content from the International Affective Picture System (IAPS) to 24 participants for 20 s. The participants reported their feelings about the pictures orally according to the self-assessment manikin on the V-A scale. In addition, they analyzed RR-intervals with PSD and classified facial expressions with SVM and Cohn–Kanade dataset. They achieved 38.0% and 54.5% in valence and arousal as a function of k, respectively.

Moreover, Huang et al. [41] proposed multi-modal emotion recognition based on facial expressions and EEG signals from a single modal to predict valence and arousal. Facial expressions were used as an external channel that involved a probability of nine facial expressions. EEG was used as an internal channel supplementing facial expressions. These two channels were fused on feature and decision levels for multi-modal emotion recognition. Owing to the lack of fusion between EEG and facial expressions, they applied a transfer learning approach for multi-task CNN architectures in a later study [42]. In EEG detection, two learning targets (valence and arousal) were detected using different support vector machine (SVM) classifiers, separately. Two decision-level fusion methods based on the enumerate weight rule or an adaptive boosting technique were used to combine facial expressions and EEG. They evaluated their method with the DEAP and MAHNOB–human–computer interface (MAHNOB-HCI) datasets and achieved 69.75% and 70.00% accuracy for the valence and arousal, respectively, and the accuracy of arousal increased by 6% after fusing them (before fusion: 69.28% and 64.00%, respectively). Similarly, other studies have predicted valence and arousal with EEG and facial expressions [24,25,43]. Rayatdoost et al. [24] predicted valence and arousal using EEG and facial expressions as the input features. They achieved accuracy of 75% and 74% for valence and arousal, respectively. Siddharth et al. [25] used EEG and facial expressions to predict valence, arousal, liking and four emotional states. They achieved accuracy of 54%. Soleymani et al. [43] focused on valence and arousal recognition. They extracted power spectral features from EEG signals and the facial fiducial points used as features to detect valence levels for each frame continuously.

Unlike using only bio-physiological signals, facial expressions can be applied to more diverse situations. Owing to the benefits of flexibility and practicality, studies are actively conducted in areas that require real-time emotion recognition in various situations. Val-Calvo et al. [26] proposed self-designed methodologies to estimate users’ emotional state in real time. They used EDA, blood volume pressure (BVP) and EEG to analyze the statistical correlation between experienced emotions and the properties of the set of features. Facial expressions were fed into a deep convolutional ensemble and trained on the FER-2013 database to obtain emotion classification. After filtering all meaningful features, they were classified by a set of eight standard classifiers into seven emotional states (neutral, disgust, happy, surprise, angry, fear and sad), achieving almost 80% accuracy. Similarly, Comas et al. [27] predicted valence, arousal, liking and seven emotional states (neutral, disgust, joy, surprise, anger, fear and sadness) using EDA, facial expressions and ECG as the input features. They trained the model with the AMIGOS and Medical Therapy datasets to predict the emotion of patients who have undergone anxiety treatment. They achieved accuracy of 64%.

### 2.4. Existing Databases

There are databases for emotion recognition, a which contain facial images [33,34,35], bio-physiological data [44,45] or both [36,37].

AffectNet [33] contains more than 1M facial images from the Internet using 1250 emotion-related keywords in six different languages. The collected images were annotated for seven discrete facial expression data and the intensity of valence and arousal. CK+ (extended version of the Cohn–Kanade database) [34] contains 593 sequences and has a total of seven emotion labels (i.e., angry, disgust, fear, happy, sadness, surprise and contempt). EMOTIC [35] consists of images and 26 discrete categories, which are labeled as the contiguous dimensions valence, arousal and dominance. EMOTIC has 23,571 images, and some images have been collected from the Google search engine.

Dreamer database [44] includes EEG and ECG signals recorded during elicited by audio-visual stimuli. In total, 23 participants self-assessed their affective state after each stimulus in terms of valence, arousal and dominance. This database could recognize emotions using SVMs. CASE dataset [45] includes eight physiological signals from the 30 participants. The participants watched various videos to elicit four affect states: amusing, boring, relaxing and scary. They were required to annotate their continuously varying emotion with a joystick. Some databases include facial images and bio-physiological signals to recognize emotion.

ASCERTAIN database [36] collected facial images and bio-physiological signals while subjects watched movie clips. After watching each clip, the subjects were required to self-report their emotional state in the form of effective rankings within 30 s. For each image, the user’s effective impression is reflected in the valence (V) and arousal (A) rate. Their bio-physiological data (i.e., ECG, EDA, frontal EEG and EMO) were also collected. This database can be used to recognize emotions. MAHNOB-HCI database [37] recorded in response to the affective stimuli. It includes face videos, audio signals, eye-gaze data and physiological signals (i.e., EDA, ECG, respiration pattern and skin temperature). They obtained the arousal, valence, dominance, predictability and emotional keywords self-reported while experimenting through two experiments.

Among the emotion recognition databases, some databases include facial image data [46,47,48] or bio-physiological signals [49] collected in driving conditions. The UTDrive DB Classic [46] collected in the context of the level of stress while 77 participants were driving in real-world urban areas highway conditions. The data contain audio, video, front car distance and driver behavior (such as CAN data and GPS). They collected UTDrive DB Portable data [46] only using smartphones without needing special equipment. The data include video, audio, GPS location and car access. The database by Ma et al. [47] classified the drivers’ emotional states into four categories (i.e., happy, bothered, concentrated and confused) and collected data while 10 drivers were driving around 24 km each. External annotators provided the emotional annotation, and the data were focused on the face video. The CIAIR database [48] collected more than 60 min of real-world driving data from over 500 drivers. Emotional annotations were not provided. The data include multi-channel video from three cameras, multi-channel audio, GPS and control signals (such as speed, engine RPM, accelerator/brake-pedal pressure and steering-wheel motion). DriveDB database [49] collected bio-physiological signals (i.e., ECG, EDA, EMG and RESP) in the context of stress level. The signals were collected while driving for 50–90 min.

### 2.5. Human-in-the-Loop Simulation

Emotion-related research has been conducted for over 100 years and has a long and extensive history. The above section introduces the previous studies centered on the emotion detection algorithm. This section introduces some of the previous studies focused on the research related to emotions considering the driving context and the emotion induction method.

Ekman [50] classified emotion into six basic emotions: anger, happiness, surprise, disgust, sadness and fear. The valence–arousal model presented by Russell [32] is a representative example of the emotion’s dimensional model. He made the *x*-axis as pleasure–misery and the *y*-axis as arousal–sleepiness and then placed various emotions on a two-dimensional plane. Jeon and Walker [51] considered that emotion needs tuning in consideration of the domain context and investigated the emotions felt by the participants in the experiment considering various driving scenarios. Accordingly, they presented nine emotions (i.e., fearful, happy, angry, depressed, curious, embarrassed, urgent, bored and relieved) considering the driving context. For research trends related to various emotions in the driving context, refer to the work by Jeon [52].

Participants must be in their emotional state to study their emotions. There are various methods to induce the desired emotion. Fakhrhosseini and Jeon [53] introduced the following methods: imagination, film, sound, music, images, reading passages, writing passages, embodiment, virtual reality, feedback, self-referent statement, social interaction, physiological manipulations, motivated performance tasks and combined techniques. They also described the details and examples of each induction method. These various induction methods have different effects. Whichever method is used, it should be considered that the desired emotion is induced and multiple emotions may be induced. It should also be considered whether the induced emotion and real emotion are the same.

## 3. Proposed Work

**Figure 1:**
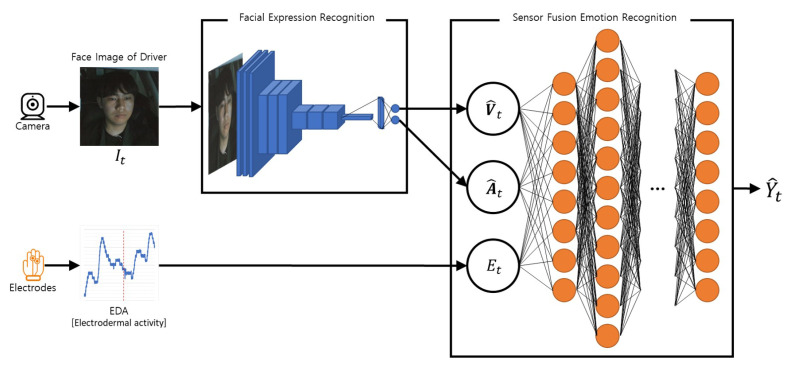
Overview of the proposed work with two major steps: FER and SFER.

We propose deep learning-based algorithms to monitor the driver’s emotional state even when emotions are not fully revealed by facial expressions while driving. Our emotion recognition system that monitors the driver’s real emotions is called DRER. We propose the two main steps to recognize the driver’s real emotion: recognizing the driver’s facial expression state and fusing bio-physiological signals with the recognized facial expression state. Figure 1 shows the proposed steps. Table 1 summarizes the terminologies used in this paper. The following subsections describe the detailed task of the individual steps.

### 3.1. Facial Expression Recognition

The most common way to express emotions is facial expressions. Therefore, the first step for the proposed work is recognizing the driver’s facial expressions. While driving, the driver’s face is recorded by a camera, and the face video becomes the input of the FER model. The FER model consists of CNN and outputs the recognized state of facial expressions in continuous represented values called valence and arousal. These representation methods can emphasize that the state of facial expressions appears continuously and includes intensity. Moreover, these mathematical representations are easy to fuse with various measured signals. Through these fusion approaches, even the emotions beyond the face can be captured.

### 3.2. Sensor Fusion Emotion Recognition

Our second step is the fusion with driver’s bio-physiological signals. As mentioned in Section 1, even if the driver’s facial expressions are detected through the FER model, they cannot be always regarded as the correct driver’s emotional states. Hence, we propose the SFER model, which consists of DNN, to recognize even the real emotions beyond the driver’s facial expressions by fusing the bio-physiological signals related to the body regulation and affected by the emotions with the output values of the FER model. The driver’s recognized emotional states are represented among several discrete categories. Section 4 describes the detailed fusion algorithms’ methodology to recognize even the driver’s real emotional states through the facial and bio-physiological information.

## 4. Methods

This section describes the detailed methodology of our algorithm for each model.

### 4.1. Facial Expression Recognition

On the basis of the deep learning algorithm, we applied end-to-end architectures to receive images (I), including the driver’s face, and output the continuous two-dimensional index, called valence $\left(\hat{V}\right)$ and arousal $\left(\hat{A}\right)$. This FER model is based on the hypothesis that the images (I), including the driver’s face, are continuously provided. To meet this hypothesis, we thoughtfully set the camera for recording the driver’s face. The details are discussed in Section 5.2.

Several preprocessing steps (e.g., resizing, normalization, detecting ROI and detecting points or movements) are required to recognize facial expressions from images [54]. However, we only have two preprocessing steps, resizing and normalizing. They are relatively simple preprocessing processes because the deep learning algorithm that we use automatically finds the region of interest and extracts features. Thus, we refer to our FER model as an end-to-end architecture. Resizing is an operation to equalize the input image’s width, height and depth with input shape (w,h,d) of the proposed deep learning algorithm. Each image pixels need to be scaled in the range from 0 to 1. This process is called normalization, which is a common technique for preprocessing for deep learning. It involves changing the values of numeric pixels in an image to a common scale without distorting the differences in the ranges of values. The equation of normalization is as follows:

(1) $$x^{\prime }=\frac{x-X_{min}}{X_{max}-X_{min}}$$

where *x* is the pixel value of an image, $x^{\prime }$ is the normalized value of the image, $X_{max}$ is the maximum pixel value of the image and $X_{min}$ is the minimum pixel value of the image. Usually, each pixel can be simply divided by 255 because most images consist of 0–255 values.

To build the FER model based on deep learning network, we benchmarked the state-of-the-art CNN models from ImageNet large-scale visual recognition challenge (ILSVRC) [55]. ILSVRC is an annual object detection and image classification competition that uses subsets from the ImageNet (a large-scale hierarchical image database) [56].

**Figure 2:**
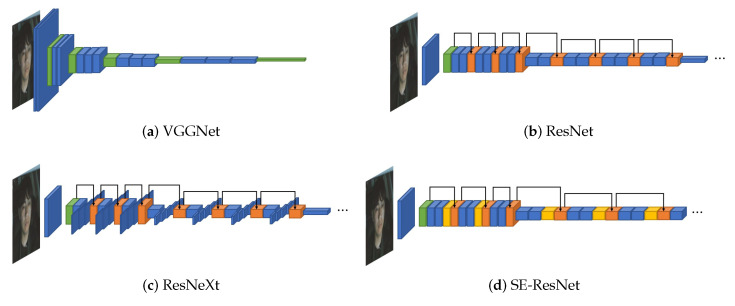
(**a**) VGGNet with vanilla CNN (blue) and max-pooling (green). (**b**) ResNet with vanilla CNN (blue), max-pooling (green), and shortcut connection (orange). (**c**) ResNeXt with vanilla CNN (blue), max-pooling (green) and shortcut connection (orange). (**d**) SE-ResNet with vanilla CNN (blue), max-pooling (green), shortcut connection (orange) and SE block (yellow).

The first model to be introduced is VGGNet [28], the 2014 ILSVRC runner-up. Although VGGNet is the runner-up, the gap between the winner is insignificant, and the structure is relatively easier to understand than the winner. Hence, we started exploring VGGNet as the underlying network. VGGNet uses multiple CNN layers to find the image’s features while reducing the width and height and deepening the input image’s depth. Figure 2a illustrates VGGNet’s structure, including the multiple CNN layers as blue boxes. In the figure, each time it passes through the green box, the max-pooling layer, it decreases width and height and increases the depth.

We also benchmarked ResNet [29], the 2015 ILSVRC winner, to build deep learning models that are deeper than VGGNet. ResNet can construct a deeper model without worrying about vanishing gradient problem by applying a shortcut connection that connects the input value to the output value for every of the two CNN layers. Its overall structure is almost similar to that of VGGNet, as shown in Figure 2b. The shortcut connections are illustrated as black arrows and the values before and after the CNN layers are summed at the orange box. The values before the CNN layers are continuously summed; thus, the amount of information to be trained is reduced. Hence ResNet can be designed deeper than VGGNet.

ResNet is a structure to make the neural network deeper, and we refer to ResNeXt [30] to search for a wider model. ResNeXt [30], the 2016 ILSVRC runner-up, adds splitting operation between the shortcut connection by applying a new dimension called a cardinality based on ResNet. ResNeXt’s splitting operation is illustrated in Figure 2c. Its overall structure is similar to that of ResNet, but every second CNN layer between shortcut connections is divided into as many boxes as the number of the cardinality based on the depth direction.

**Figure 3:**
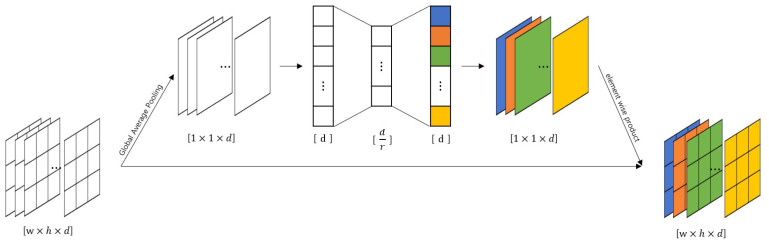
Illustration of the SE block; different colors represent each different channel.

We applied the SE block proposed by Roy et al. [57]. The SE block inserted in the middle of the model corrects the weights between the feature map’s channels that is the intermediate result. In Figure 3, the feature maps entered as the SE blocks are converted into values representing each channel through global average pooling. These representative values are recalibrated as it passes through the bottleneck structure formed by the reduction ratio *r*. The recalibrated values are applied to the existing feature map element-wise. SENet, which applies the SE block to the existing model, is the 2017 ILSVRC winner. We applied the SE block to the right before the summation layers of ResNet and ResNeXt. The model with the SE block applied to ResNet is illustrated in Figure 2d, and the SE blocks are shown as yellow boxes before the orange boxes.

Every backbone network introduced above has 1000 units of final fully connected hidden layers to classify the 1000 different object categories; in our FER model, we modified the final hidden layer to have two units of a fully connected layer to output the recognized state of facial expressions in two continuous represented values, valence and arousal. Then, the tanh function transforms a vector of two output values of the fully connected layer into recognized valence and arousal values. The tanh turns them into values between −1 and 1. The absolute value of the transformed valence represents the degree, where a positive value represents the degree of attractiveness and a negative value represents the degree of aversiveness. The numerical size of the transformed arousal value represents the perceived intensity. For instance, the negative arousal values have lower perceived strength than the positive arousal values. The reason that the output of the FER model is represented in the continuous values is discussed in Section 4.2. Algorithm 1 summarizes detailed procedures of FER.
**Algorithm 1** Our Facial Expression Recognition (FER) Algorithm.**Require:** The face image of driver at time *t*: $I_{t}$**Ensure:** The recognized valence and arousal level of driver by facial expressions at time *t*: $\left({\hat{V}}_{t},{\hat{A}}_{t}\right)$   **Initialize:**      Let the facial expression recognition model be FER_Model      Let FER_Model be one of VGGNet, ResNet, ResNeXt, SE-ResNet, and SE-ResNeXt      Let final hidden layer of FER_Model be fully connected layer which have 2 units and tanh function as activation function   **while** Driving **do**      # Resizing      Resize $I_{t}\in Z^{w\times h\times d}$      # Normalization      $I_{t}=\frac{I_{t}-min\left(I_{t}\right)}{max\left(I_{t}\right)-min\left(I_{t}\right)}$      $({\hat{V}}_{t},{\hat{A}}_{t})=$FER_Model($I_{t}$)   **end while**

### 4.2. Sensor Fusion Emotion Recognition

To classify the driver’s real emotions $\left(\hat{Y}\right)$ that are not fully revealed on the face, we applied a DNN-based supervised learning architecture that fused our output values of the FER model and bio-physiological signals to output the most confident emotion among several discrete categories. Although these discrete representations allowed simplification and easy understanding of the recognized emotion, confirmatory biases and priming effects depend on the individual’s experience [58,59]. Hence, it is important to define the appropriate driver’s emotion category (C) for simplified emotion that gives accurate intuition.

**Figure 4:**
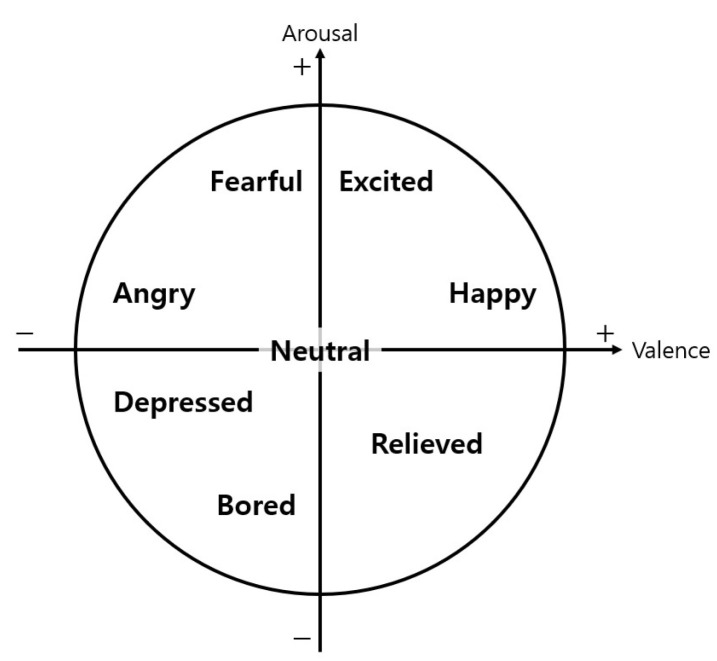
The correlations between the valence and arousal of defined eight emotions.

Here, we used the arousal–valence model proposed by Russell as the basic frame. By considering the nine emotions that considered the driving context [51] and the emotions extracted from the AffectNet results, we investigated the following eight emotions: neutral, happy, excited, fearful, angry, depressed, bored and relieved. Arousal and valence are both 0 for neutral and both positive for happy and excited. Arousal is positive but valence is negative for fearful and angry. Arousal and valence are both negative for depressed and bored. Valence is positive and arousal is negative for relieved. Figure 4 shows the correlations between the valence and arousal of all eight emotions. Through the aforementioned process, we set the number of emotion categories (*k*) to eight and defined the emotional state categories for the driver (C), as shown in Equation (2).

(2) $$C=\left[\begin{array}{c} C_{1}\\ C_{2}\\ C_{3}\\ C_{4}\\ C_{5}\\ C_{6}\\ C_{7}\\ C_{8} \end{array}\right]=\left[\begin{array}{c} \mathrm{Neutral}\\ \mathrm{Happy}\\ \mathrm{Excited}\\ \mathrm{Fearful}\\ \mathrm{Angry}\\ \mathrm{Depressed}\\ \mathrm{Bored}\\ \mathrm{Relieved} \end{array}\right]$$

As mentioned in Section 2.2 and Section 2.3, there are various bio-physiological signals to recognize human emotion, but we select some representative signals in consideration of the vehicle environment and industrial demand. Although an EEG is widely used, expensive medical equipment is required for its measurement. Some of the popular signals are cardiac signals (e.g., ECG or PPG). However, because noise caused by the movement of a subject is easily generated during the measurement of the cardiac signals [17], they are not suitable considering the driving environment. Therefore, among the many bio-physiological signals affected by the emotional experience, we selected EDA considering two aspects. The first aspect is how much it relates to emotions. According to several related studies, EDA contains the most emotional information [54]. Healey and Picard [49] and Deng et al. [14] showed that EDA is the most representative signal for combination to capture the stress of drivers. The second aspect is the comfort to measure while driving. Cardiac signals are usually measured with electrodes attached to the chest. A driver cannot drive with electrodes on his chest all the time. Although EDA signals are also measured with electrodes, they can be measured on the hand palm. Moreover, recently, they can be measured in dry conditions on a location that has less interference while driving (e.g., wrist) [60]. Hence, we proposed the EDA signal (E) and the outputs of the FER $\left(\hat{V},\hat{A}\right)$ as the input features (X) to detect and classify the driver’s real emotion during driving.

Before feeding input features into the deep learning architecture, the SFER model also needs preprocessing to input features (X). Cohn et al. [61] presented that individual differences in facial expressions were moderately strong. Naveteus and Baque [62] presented individual differences in EDA. These individual differences are overcome by personalization work through mean subtraction. Personalization needs to be performed individually. Each individual’s mean value is subtracted from each feature to capture the neutral valence’s baseline, arousal and EDA. Then, every input feature is set to 0–1 through normalization because the personalized input features have different scales. Each feature must be performed by subtracting each sample’s minimum value and dividing it with the range between maximum and minimum values, as shown in Equation (1). Personalization and normalization are possible because input feature (X), especially valence and arousal values $\left(\hat{V},\hat{A}\right)$ which are from the FER, rely on continuous representation.

We built a DNN, the most fundamental deep learning model, with multiple layers between input and output. Even though there are many advanced deep learning networks for solving difficult tasks, we already secured the FER feature vector $\left(\hat{V},\hat{A}\right)$, extracted from the state-of-the-art deep learning model and the EDA feature vector (E) selected on the basis of the related studies. Hence, if there are high-quality data for supervised learning, it is possible to classify the driver’s emotional state with only the DNN model through the deep learning training process. The DNN model consists of *N* and maximum *M* units of hidden layers. We set the number of final layer units to *k* and outputs a vector of size *k* to represent one of the *k* defined emotions. The output vector pass for the activation function called softmax to represent the confidence probability for each emotion. The DNN model was trained and tested on simulated data, as described in Section 5.2. Algorithm 2 summarizes the detailed procedures in recognizing the driver’s emotional state from the valence $\left(\hat{V}\right)$, arousal $\left(\hat{A}\right)$ and EDA (E) values.
**Algorithm 2** Our Sensor Fusion Emotion Recognition (SFER) Algorithm.**Require:** Variable for driver distinguish(Driver ID): ID   List of defined emotional state categories for driver: *C*   The recognized valence and arousal level of driver by facial expressions at time *t*: $\left({\hat{V}}_{t},{\hat{A}}_{t}\right)$   The measured EDA response of driver at time *t*: $E_{t}$**Ensure:** The recognized real emotion of driver at time *t*: ${\hat{Y}}_{t}$   **Initialize:**      Let the sensor fusion emotion recognition model be SFER_Model      Let SFER_Model be the DNN      Let final hidden layer of SFER_Model be fully connected layer which *n* units and softmax function as activation function      ${\hat{V}}^{ID}=\left[\right],\;{\hat{A}}^{ID}=\left[\right],\;E^{ID}=\left[\right]$   **if** Accumulated data for the driver with ID exists **then**      Load ${\hat{V}}^{ID},{\hat{A}}^{ID},E^{ID}$   **end if**   **while** Driving exists **do**      # Accumulate data per each individual for personalization      ${\hat{V}}^{ID}.insert\left({\hat{V}}_{t}\right)$,   ${\hat{A}}^{ID}.insert\left({\hat{A}}_{t}\right)$,  $E^{ID}.insert\left(E_{t}\right)$      # Mean subtraction      $\left({\hat{V}}_{t},\;{\hat{A}}_{t},\;E_{t}\right)-=\left(mean\left({\hat{V}}^{ID}\right),\;mean\left({\hat{A}}^{ID}\right),\;mean\left(E^{ID}\right)\right)$      # Normalization      $X_{t}^{T}=\left(\frac{{\hat{V}}_{t}-min\left({\hat{V}}^{ID}\right)}{max\left({\hat{V}}^{ID}\right)-min\left({\hat{V}}^{ID}\right)},\frac{{\hat{A}}_{t}-min\left({\hat{A}}^{ID}\right)}{max\left({\hat{A}}^{ID}\right)-min\left({\hat{A}}^{ID}\right)},\frac{E_{t}-min\left(E^{ID}\right)}{max\left(E^{ID}\right)-min\left(E^{ID}\right)}\right)$      $\hat{Y_{t}^{𝕝}}=$SFER_Model($X_{t}$)      ${\hat{Y}}_{t}=C\left[argmax\left(\hat{Y_{t}^{𝕝}}\right)\right]$   **end while**

## 5. Experiments

This section presents the experimental evaluations of the proposed algorithms. We performed our experiments on a machine with Intel Core i9-9980XE CPU at 3.00 GHz, 125 GB RAM and Nvidia Titan RTX GPU. We applied the state-of-the-art CNN models from ILSVRC to our FER models and we used AffectNet [33] database to train and evaluate our FER models. The details are described in Section 5.1. Section 5.2 describes the experimental design of the human-in-the-loop simulation for collecting datasets. These datasets contain driver’s facial images, EDA measurements and induced emotions as the ground truth emotion labels. We used these data to train and evaluate the SFER models. More details are presented in Section 5.2.

### 5.1. Facial Expression Recognition

**Figure 5:**
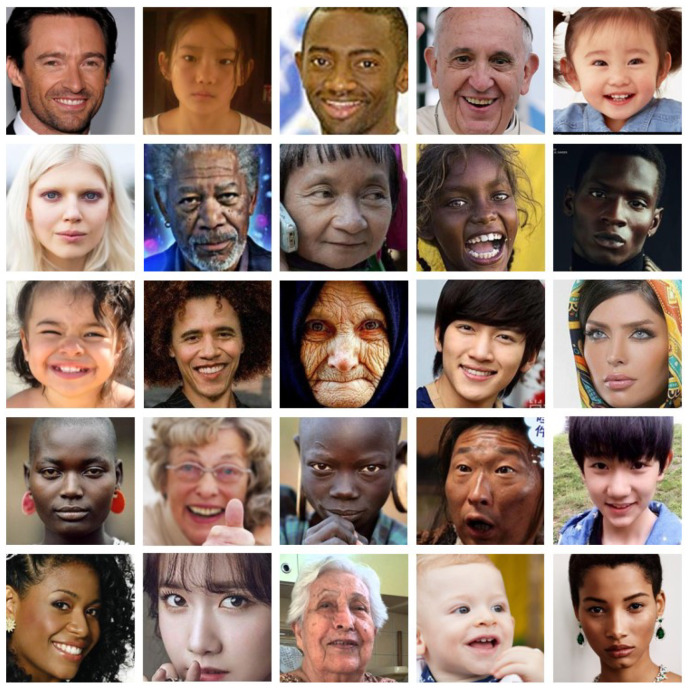
Sample images in the AffectNet, including faces of people of different races, ages and gender.

As discussed above, we used AffectNet [33], one of the largest databases for facial expressions, to train and evaluate our FER models. It contains approximately 1M facial images collected in the wild and annotation information for the FER. AffectNet contains a significant number of emotions on faces of people of different races, ages and gender. Figure 5 shows sample images in the AffectNet. Moreover, the biggest reason we used AffectNet is that it contains manually annotated intensity of valence and arousal. We only used manually annotated images containing 320,739 training samples and 4500 validation samples, excluding uncertain images. Unfortunately, AffectNet has not yet released the test samples. Hence, we compare the validation set results between their baseline methods in Section 6.1.

We proposed various FER models for a comparative experiment. Every proposed FER model, evaluated in this study, consists of input shape (224,224,3), several CNN layers and one fully connected layer as the trainable layers. Because we used RGB images, we set the depth of the input image to 3; however, the depth of the input image can be changed to 1 when using the binary images from the NIR camera to secure more robustness against changes in illuminance, as proposed by Gao et al. [5]. In the following, we distinguished the models by their base architectures and number of trainable layers. To validate the effectiveness according to the model’s depth, we designed the models with different depths using VGGNet [28] and ResNet [29]. To validate the performance of the parallelization and the channel-wise attention of CNN layers, we applied ResNeXt [30] and SE block [31] to our FER models. All of our FER models are described as follows, and detailed configurations of each structure are outlined in Table 2, one per column.

- VGG14 is based on VGGNet architectures and consisted of 13 CNN layers. The last three fully connected layers are replaced with one fully connected layer with only two units, to represent valence and arousal, respectively. The model has 14 trainable layers, thus it is called VGG14.
- VGG17 is based on VGGNet, and three more CNN layers are added to VGG14. It consists of 16 CNN layers and 1 fully connected layer.
- ResNet18 is based on ResNet and has 18 trainable layers (17 CNN layers and 1 fully connected layer). Compared with VGG17, there is only one more CNN layer; however, ResNet18 has the shortcut connection for every two CNN layers, except the first CNN layer. The layers between shortcut connections are represented as curly brackets in Table 2.
- ResNet34 is based on ResNet and has 34 trainable layers (33 CNN layers and 1 fully connected layer). It also has shortcut connections for every two CNN layers, except the first CNN layer. The layers between shortcut connections are represented as curly brackets in Table 2.
- ResNet50 is based on ResNet and has 50 trainable layers (49 CNN layers and 1 fully connected layer). It has a shortcut connection for every three CNN layers, except the first CNN layer. The layers between shortcut connections are represented as curly brackets in Table 2.
- ResNet101 is based on ResNet and has 101 trainable layers (100 CNN layers and 1 fully connected layer). It also has a shortcut connection for every three CNN layers, except the first CNN layer. The layers between shortcut connections are represented as curly brackets in Table 2.
- ResNeXt34 is based on ResNeXt, and it is composed of 34 trainable layers (33 CNN layers and 1 fully connected layer). The cardinality is set to 32. The last CNN layers between shortcut connections are propagated by splitting them into 32 on a channel basis. In Table 2, the shortcut connections are represented as curly brackets, and the splitting operation is represented as every last layer in curly brackets.
- SE-ResNet34 applies the SE block to ResNet34. The SE blocks are positioned between the last CNN layers of shortcut connections and the merge points of the shortcut connections, as shown in Table 2. The detailed structure is shown in Figure 3, and the reduction ratio *r* is set to 4.
- SE-ResNeXt34 applies the SE block to ResNeXt34. The SE blocks are positioned between the last CNN layers of shortcut connections and the merge points of the shortcut connections, as shown in Table 2. The structure is the same as the SE block of SE-ResNet34, and the reduction ratio *r* is also set to 4.

We trained our FER models to minimize the distance between the predicted (${\hat{V}}_{i},{\hat{A}}_{i}$) and true ($V_{i},A_{i}$) values of the valence and arousal using AffectNet [33]. L2 loss function measures the distance and is shown as follows:

(3) $$L\left(\hat{V},\hat{A},V,A\right)=\frac{1}{2n}\sum_{i=1}^{n}\left({\left({\hat{V}}_{i}-V_{i}\right)}^{2}+{\left({\hat{A}}_{i}-A_{i}\right)}^{2}\right)$$

where *n* is the number of training samples, ${\hat{V}}_{i}$ is the predicted valence value of *i*th training sample, $V_{i}$ is the true valence value of *i*th training sample, ${\hat{A}}_{i}$ is the predicted arousal value of *i*th training sample, and $A_{i}$ is the true arousal value of *i*th training sample. We used the Adam algorithm [63], a popular optimizer, to optimize the model parameters. We set the learning rate to 0.001 and the first and second moments to 0.9 and 0.999, respectively. We tried to train over 10 epochs (over 3,207,390 iterations), and the training was terminated when the loss value on the validation set was stable. To compare our models, we used root mean squared error (RMSE) on the validation set:

(4) $$RMSE=\sqrt{\frac{1}{m}\sum_{i=1}^{m}{\left({\hat{y}}_{i}-y_{i}\right)}^{2}}$$

where *m* is the number of validation samples, ${\hat{y}}_{i}$ is the predicted value of *i*th validation sample and $y_{i}$ is the true value of *i*th validation sample. The RMSE values of valence and arousal are compared separately.

### 5.2. Sensor Fusion Emotion Recognition

Thirteen volunteers (six men and seven women) participated in this study, five times per participant. The experiment had to be conducted by inducing eight emotions for all participants; hence, it was impossible to conduct all experiments in a single day. We experimented by grouping two similar emotions (2 emotions/session × 4 session = 8 emotions). One session was conducted as a pretest. All experiments were conducted after obtaining approval from Kookmin University’s IRB (KMU-202005-HR-235).

**Figure 6:**
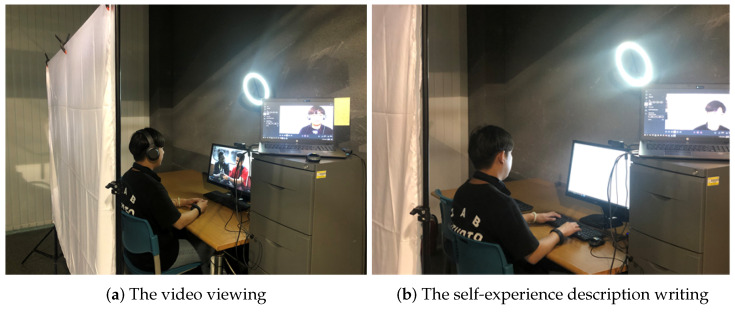
(**a**) The participants’ emotions are induced through video viewing. (**b**) The participants describe their own experiences related to the emotions induced.

To study the eight emotions defined in Section 4.2, we needed to induce the participants into each emotion situation or state. We applied a technique that combines film watching and writing passages, as shown in Figure 6. After watching a 4–6 min video to induce the desired emotion, the researchers asked 70 people who are not familiar with our study to watch the video online and gathered their opinion about emotional state after viewing. After confirming that the emotion was induced as intended, the video was used in this experiment. To increase the emotions’ duration and reinforce the emotions induced through the video, we asked the participants to freely describe for 13 min their own experiences related to the emotions induced. The video viewing and self-experience description are two of the most valid emotion induction and reinforcement techniques [53]. During video viewing and self-experience description, we recorded the driver’s facial image and measured his/her EDA. After that, the participants’ self-reported emotions were asked through a survey. Then, the driving was carried out for 5 min in the driving simulator. We recorded the driver’s face image and measured his/her EDA. After finishing the driving, the experimenters debriefed the purpose of the study. In other words, by neutralizing the participants’ emotions, we made sure that the participants’ moods are close to the baseline level when they are leaving the laboratory.

**Figure 7:**
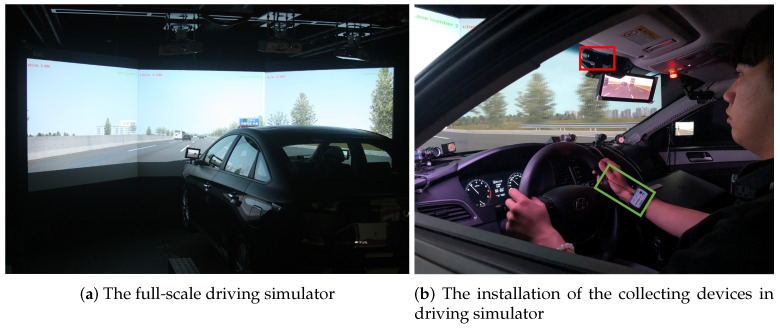
(**a**) Three-channel projectors and screens and the cabin of the full-scale driving simulator. (**b**) The camera is installed between the windshield and the headliner (red) and the biomedical instrument is set on the driver’s wrist for EDA (green).

In the experiment, we used a full-scale driving simulator with six DOF motion base equipped with AV Simulation’s SCANeR Studio 1.7 (AVSimulation, Boulogne-Billancourt, France, https://www.avsimulation.com/ (accessed on 18 March 2021)). The LF Sonata, a Hyundai midsize sedan, was utilized as a cabin. Three-channel projectors and three 2080 mm × 1600 mm screens were connected horizontally to visualize the driving scene. The participants’ physiological signals of EDA was collected using a BioPac bioinstrument (BIOPAC Systems, Inc., Goleta, CA, USA, https://www.biopac.com/ (accessed on 18 March 2021)). The bioinstrument guarantees excess 70 dB of signal to noise ratio (SNR). To acquire a reliable EDA signal, we removed the dead skin cells on the hand to prevent the interruption of signal collection and applied an isotonic electrode paste to the electrode for increasing accuracy. In addition, before starting all experiments, we observed the EDA waveform to confirm that there is no visible noise throughout the signal. For the driver’s face image, we used BRIO 4K (Logitech, Lausanne, Switzerland, https://www.logitech.com/, accessed on 18 March 2021) with 720 × 720 pixel and 30 fps for video viewing, self-experience description and in the driving simulator. Figure 7a shows the full-scale driving simulator. Figure 7b shows the installation of the BioPac bioinstrument and the camera in the driving simulator while the driver is driving. The camera was installed between the windshield and the headliner in front of the sun visor to avoid that driver’s face being partially occluded by the steering wheel or hand.

Among the data collected through simulation, the video and EDA data collected while driving were used for training and evaluating the SFER model and the rest of the data were used for reference. The driving data were acquired while each volunteer was driving for about 5 min per each emotion. The driver’s facial expressions images were acquired with a 30 Hz sampling rate, and the EDA data were acquired when considering a 100 Hz sampling rate. The acquired driving data cannot be used for training of the FER models. To train the FER models, the true valence and arousal values are required for each facial image, but the driving data involve the induced emotion as the ground truth label.

To validate the effectiveness of input features and model structure, we proposed various SFER models. Each proposed SFER model, evaluated in this study, consists of fully connected layers as the input layer, output layer and multiple hidden layers. Every output vector from the hidden layers passes through the ReLU activation function, and the output vector from the output layer passes through the softmax activation function. In the proposed SFER model, we set the number of output layer units to 8 because the number of emotional state categories for driver (k) is defined in eight categories, as described in Section 4.2. The models are distinguished by their input features, several hidden layers (L) and several maximum units (U) of hidden layers. In the following, the front part of the model name means the kind of input features. If the front part of the model name is VA or E, the model uses only the output value of the FER model $\left(\hat{V},\hat{A}\right)$ or the EDA (E) value as an input. If the model name starts with VAE, both of the output value of the FER model $\left(\hat{V},\hat{A}\right)$ and EDA (E) value are used as input values. The number in parentheses of the model name means the number of hidden layers and maximum units as (L,U). All the proposed SFER models are described as follows. Table 3 presents their detailed configurations.

- E(3,64): This only uses EDA (E) value measured as the input value. The number of input layer units is 1, and this SFER model recognizes the driver’s emotional state with only bio-physiological information. The number of hidden layers is 3 and the number of maximum units is 64.
- E(8,512): This only uses EDA (E) value measured equal to E(3,64). However, the number of layers and maximum units of the model are made deeper and wider than E(3,64). The number of hidden layers is 8 and the number of maximum units is 512.
- VA(3,64): This only uses valence $\left(\hat{V}\right)$ and arousal $\left(\hat{A}\right)$ values from the FER model as input values. The number of input layer units is 2, and this SFER model recognizes the driver’s emotional state with only the FER information. The number of hidden layers is 3 and the number of maximum units is 64.
- VA(8,512): This only uses valence $\left(\hat{V}\right)$ and arousal $\left(\hat{A}\right)$ values equal to VA(3,64). However, the number of layers and maximum units of the model are made deeper and wider than VA(3,64). The number of hidden layers is 8 and the number of maximum units is 512.
- VAE(3,64): This uses valence $\left(\hat{V}\right)$, arousal $\left(\hat{A}\right)$ and EDA (E) values as input values. The number of input layer units is 3, and this SFER model recognizes the driver’s emotional state with both FER and bio-physiological information. The number of hidden layers is 3 and the number of maximum units is 64.
- VAE(8,512): This uses valence $\left(\hat{V}\right)$, arousal $\left(\hat{A}\right)$ and EDA (E) values equal to VAE(3,64). However, the number of layers and maximum units of the model are made deeper and wider than VAE(3,64). The number of hidden layers is 8 and the number of maximum units is 512.
- VAE(9,1024): This uses valence $\left(\hat{V}\right)$, arousal $\left(\hat{A}\right)$, and EDA(E) values as input values. The number of hidden layers is 9 and the number of maximum units is 1024.
- VAE(9,2048): This uses valence $\left(\hat{V}\right)$, arousal $\left(\hat{A}\right)$ and EDA (E) values as input values. The number of hidden layers is 9 and the number of maximum units is 2048.
- VAE(10,1024): This uses valence $\left(\hat{V}\right)$, arousal $\left(\hat{A}\right)$ and EDA (E) values as input values. The number of hidden layers is 11 and the number of maximum units is 1024.

**Figure 8:**
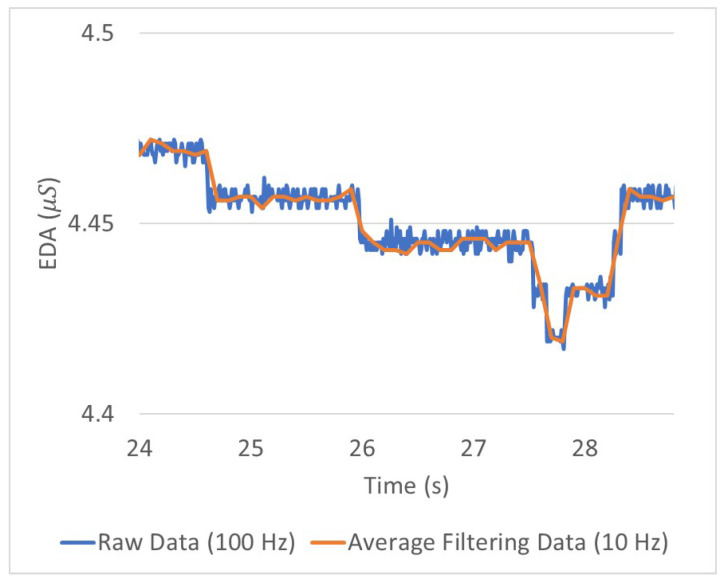
Part of raw and average filtering data of measuring EDA electrical conductance while one of simulation driving.

The minimum vehicle control cycle required by the industry is 10 Hz. To satisfy this requirement, all the proposed SFER models have a 10 Hz recognition frequency. The time window of input data was set to 0.1 s because the driver’s emotions can change in the same period according to the vehicle control state that changes at least 10 Hz in a period. Hence, to train and evaluate these proposed SFER models, filtering was required for each input datum, $\hat{V}$, $\hat{A}$ and *E*. The output value of the FER model $\left(\hat{V},\hat{A}\right)$, which has valence and arousal value per input image, has a sampling rate of 30 Hz. Therefore, the filtering value was calculated as the average value of three valence and arousal values for the previous 0.1 s in every 10 Hz. Because when the EDA data were acquired a 100 Hz sampling rate was considered, the filtering value of the EDA value (E) was calculated as the average value of ten EDA values for the previous 0.1 s in every 10 Hz. The average filtering reduces the fine residual noise remaining in the EDA waveform, as shown in Figure 8.

Through the average filtering with a time window of 0.1 s, the total number of input data $\left(\hat{V},\hat{A},E\right)$ in which one valence, one arousal and one EDA value set is observed is 310,389. The data were divided into training and test sets at an 8:2 ratio; 20% of the training set was used for validation to prevent overfitting. Hence, we trained the SFER model with a training and validation set containing 198,610 and 49,653 data, respectively. We evaluated the trained SFER model with a test set involving 62,066 data.

The proposed SFER models require all input and output to be numeric because they operate by a series of numerical operations from input to output. This means that the driver’s defined emotional state categories (C) must be converted to a numerical form, and the SFER models’ output value $\left({\hat{Y}}^{𝕝}\right)$ is needed to be converted back into the categories (C). One-hot encoding, which is the most widespread approach for this conversion, creates a separate binary column for each possible category and inserts 1 into the corresponding column. The converted categories $\left(C^{𝕝}\right)$ is shown as follows:

(5) $$\begin{array}{c} {C_{1}^{𝕝}}^{T}=\left[1,0,0,0,0,0,0,0\right]\\ {C_{2}^{𝕝}}^{T}=\left[0,1,0,0,0,0,0,0\right]\\ {C_{3}^{𝕝}}^{T}=\left[0,0,1,0,0,0,0,0\right]\\ \vdots \;\;\\ {C_{8}^{𝕝}}^{T}=\left[0,0,0,0,0,0,0,1\right] \end{array}$$

where k=8, as defined in Section 4.2. Then, induced emotion to the driver (Y) composed of *C* is also converted into $Y^{𝕝}$, comprised of $C^{𝕝}$. Hence, we can find the numerical cross-entropy loss between the induced emotion ($Y^{𝕝}$) and predicted emotion $\left({\hat{Y}}^{𝕝}\right)$:

(6) $$L\left({\hat{Y}}^{𝕝},Y^{𝕝}\right)=-\frac{1}{n}\sum_{i=1}^{n}\left({Y_{i}^{𝕝}}^{T}\times log\left({\hat{Y}}_{i}^{𝕝}\right)\right)$$

where *n* is the number of training samples, ${\hat{Y}}_{i}^{𝕝}$ is the one-hot encoded predicted emotion of the *i*th training sample and $Y_{i}^{𝕝}$ is the one-hot encoded induced emotion of the *i*th training sample. The sum of all elements of ${\hat{Y}}_{i}^{𝕝}$ is 1 because the output vector passed through softmax activation function to predict. We used the Adam algorithm [63], the same as the FER model training, to optimize the parameter of our proposed SFER models. We set the learning rate to 0.001 and the first and second moments to 0.9 and 0.999, respectively. We tried to train over 30 epochs (over 5,958,300 iterations), and the accuracy evaluation of each model was performed when the loss value on the validation set reached stable point. In order for trained model to output the recognized emotional state, we obtained the index with the largest value of ${\hat{Y}}^{𝕝}$ and converted it to the emotional category of the corresponding index among the *C*. Through this conversion, ${\hat{Y}}^{𝕝}$ was converted back into the recognized real emotional state ($\hat{Y}$), comprised of *C*. We compared the SFER models’ accuracy through the correctly recognized ratio with $\hat{Y}$ and *Y*.

## 6. Results

In this section, we present the experiment results of the proposed algorithms. In Section 6.1, the results of training and comparison of the proposed FER models are described. The results of training and comparison of the proposed SFER models are described in Section 6.2. After analyzing the results, the DRER algorithm that we finally proposed is constructed by combining the FER model and SFER model with the best performance. The performance of DRER algorithm when compared with the state-of-the-art algorithms is also presented in Section 6.2.

### 6.1. Facial Expression Recognition

**Figure 9:**
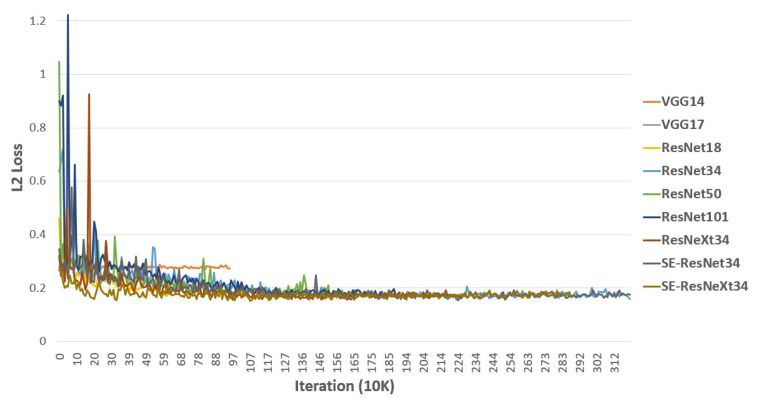
L2 loss function on the validation set of the proposed FER model training.

Figure 9 shows the value of validation loss over the 3M iterations for all our proposed FER models. Some of the training was stopped early if the validation loss had plateaued.

To validate the effectiveness of the network depth, we compared the RMSE values of valence and arousal on the validation set between models based on VGG and ResNet, as shown in Table 4. If we compared VGG14 and VGG17 results, we expected that VGG17 obtains lower RMSE values because it is a deeper network than VGG14. However, in Table 4, VGG17 has larger RMSE values of both valence and arousal than those of VGG14. This degradation has already been reported in several studies. The RMSE values will degrade on the model based on ResNet because ResNet is the architecture that overcomes this limitation. As expected, ResNet34’s RMSE values of valence and arousal are 0.418 and 0.378, respectively, which are much lower than those of VGG14. However, looking at the results of ResNet50 and -101, which have deeper networks than ResNet34, the degradation problem is not completely resolved. Even though the models are based on ResNet, the RMSE values increase when the number of trainable layers is deeper than 34. To validate the parallelization performance, we compared ResNeXt34 with ResNet34, which showed the best performance among the proposed models with different depths.

Table 4 shows that ResNeXt’s RMSE values of valence and arousal are 0.146 and 0.372, respectively. Increasing the number of layers did not lower the RMSE values; however, better recognition performance was obtained by splitting some of the CNN layers to make them parallel. To validate the channel-wise attention performance, we also compared ResNet with SE-ResNet and ResNeXt with SE-ResNeXt. The results of these models of valence and arousal are 0.419 and 0.377, respectively, as shown in Table 4. The SE-ResNet34 result shows that the RMSE value of arousal decreased compared with that of ResNet34, but, as the RMSE value of arousal decreased, the RMSE value of valence increased. From the SE-ResNet34 result alone, we cannot say that SENet is effective. However, the SE-ResNeXt34 result shows a decrease of RMSE value of valence from ResNeXt34 with a minute change of arousal’s RMSE value. Hence, SENet can be considered to be effective for the FER. SE-ResNeXt34 shows the best performance (0.408 and 0.373 of valence and arousal, respectively) in recognizing the facial expressions as valence and arousal states among all of the proposed models. It is an equivalent result compared with the baseline method proposed by Mollahosseini et al. [33], in which the RMSE values of valence and arousal are 0.37 and 0.41, respectively. Although the performance for valence and arousal tend to be opposite between each method, they are on the same level overall. The baseline method has two separate models that output the valence and arousal states. While the input face image needs to be cropped, our proposed models output the valence and arousal states at once without cropping. Thus, the proposed SE-ResNeXt34 is a better FER model than the baseline method.

### 6.2. Sensor Fusion Emotion Recognition

**Figure 10:**
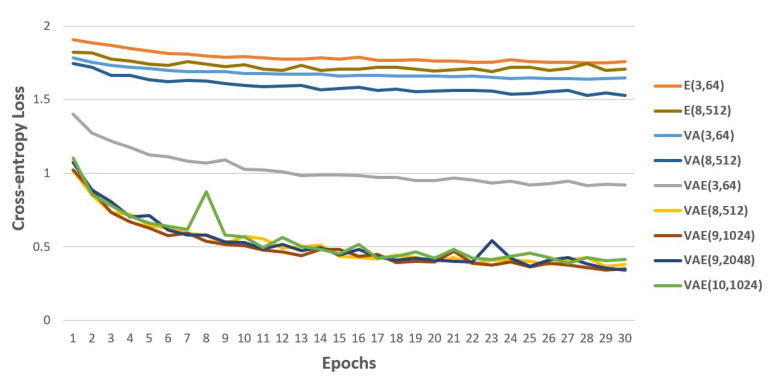
Cross-entropy loss on validation set of the proposed SFER model training.

Figure 10 shows the value of validation loss over the 30 epochs for all our proposed SFER models. We found the performance of fusing with bio-physiology signals through the comparison with VA(3,64), E(3,64) and VAE(3,64). The validation loss of VAE(3,64) is lower than those of VA(3,64) and E(3,64) on every epoch.

Table 5 presents the accuracy on test set. In the table, the accuracy of VAE(3,64) is 75% higher than that of VA(3,64) and 99% higher than that of E(3,64). The same experiment was compared with deeper and wider models: the accuracy of VAE(8,512) is 114% higher than that of VA(8,512) and 146% higher than that of E(8,512). A more interesting point is the rate of increase in accuracy for the model with fused inputs as the model structure gets more complex. As the model structure using only bio-physiology information has become deeper and wider, from E(3,64) to E(8,512), the accuracy has increased by 8%. The accuracy of VA(8,512) using only facial information has increased by 9% from VA(3,64). The accuracy of VAE(8,512) fusing facial and bio-physiology information has increased by 34% from VAE(3,64). On the basis of these results, we proved the effectiveness of fusing the recognized facial expressions and the driver’s measured EDA information to recognize the driver’s real emotion.

To find the model with the best accuracy while fusing both information, we compared the result for various structures of VAE models, as shown in Table 5. As the number of layers increased to 9 and the maximum number of units increased to 1024, the accuracy improved continuously. Then, the accuracy of VAE(9,1024) is 0.886. However, after VAE(9,1024), the accuracy of VAE(9,2048), which has the maximum number of units two times that of VAE(9,1024), is 0.865. Similarly, the accuracy of VAE(10,1024), which has one more layer than VAE(9,1024), is 0.871. Both models have lower accuracy than the that of VAE(9,1024). Hence, the accuracy does not continue to increase as the model gets deeper and wider. The proposed SFER model showed the highest accuracy of 0.886 with VAE(9,1024).

Table 6 is the confusion matrix of the evaluation result of VAE(9,1024), which achieves the best accuracy among the proposed SFER models. It shows the recognition rate for each induced emotions. The highest recognition rate is 0.930 for the “happy” emotion, and the lowest recognition rate is 0.861 for the “depressed” emotion. Every recognition rate is between 0.861 and 0.930. Thus, the proposed SFER model can recognize evenly without bias to any emotion.

**Figure 11:**
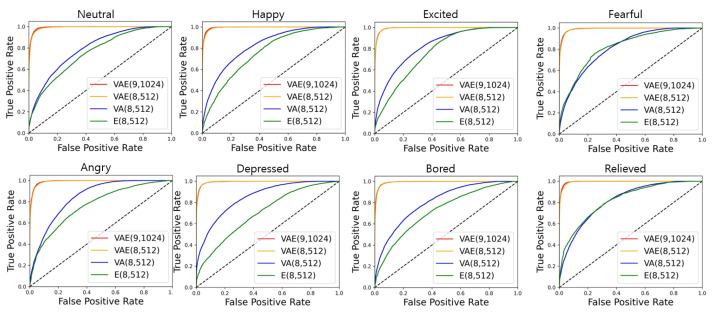
ROC curve for each defined emotion.

The receiver operating characteristic (ROC) curve is a plot with a true positive rate (TPR) on the y-axis against a false positive rate (FPR) on the x-axis. Figure 11 presents the ROC curve using one-versus-rest method for each emotion on the test set. It involves eight graphs, including VAE(9,1024), VAE(8,512), VA(8,512) and E(8,512) ROC curves to compare the accuracy depending on the input features. There was little difference between VAE(9,1024) and VAE(8,512) on the ROC curve, which is located above VA(8,512) and E(8,512). Although VA(8,512) was higher on the ROC curve than E(8,512) in the case of most emotions, with respect to relieved and fearful, there was little difference between VA(8,512) and E(8,512).

Table 7 shows the values of area under the curve (AUC) of the ROC curves in Figure 11 according to the emotions considering in each model. The AUC of classification models with good performance is close to 1, which means it has a good measure of separability. As shown in Table 7, the average AUCs of VAE(9,1024) and VAE(8,512) were 0.994, which was 20% and 30% higher than those of VA(8,512) and E(8,512), respectively. VAE(9,1024) and E(8,512) had high AUC for all emotions, whereas VA(8,512) had the lowest AUC at 0.790 for neutral and the highest AUC at 0.848 for depressed. E(8,512) had the lowest AUC of 0.705 at depressed and the highest AUC of 0.826 at relieved.

Based on the above experiment results, we realized the best FER model, SE-ResNeXt, and the best SFER model, VAE(9,1024). Therefore, we proposed the DRER algorithm that recognizes the driver’s real emotion while driving by combining SE-ResNeXt and VAE(9,1024). The performance of the DRER algorithm in comparison with other state-of-the-art algorithms is shown in Table 8. A good emotion recognition model should have the following qualities: high accuracy and various emotional states. Although Machot et al. achieved high accuracy using only bio-physiological signal, they only classified four emotional states [22]. Comas et al. classified seven emotional states by fusing the facial expressions and bio-physiological signals, but the accuracy was 64% [27]. On the other hand, the DRER algorithm achieved a high emotion recognition accuracy of 0.89 for eight different emotional states using facial expressions and EDA as the input features. The DRER algorithm we proposed shows the highest accuracy while classifying the most emotional states.

## 7. Conclusions and Future Work

In this paper, we propose the DRER algorithm based on deep learning algorithm to recognize the driver’s emotional state that does not always appear clearly on their face while driving. On the basis of CNN, we propose the FER model, an end-to-end architecture, to recognize the driver’s facial expression state through the driver’s face image photographed without any additional work. Then, on the basis of DNN, we propose the SFER model to recognize the driver’s real emotional state, which is not fully revealed on the face, by fusing the recognized facial expression state and the driver’s EDA signals. We define the appropriated driver’s emotion categories, and the output of our proposed SFER model is represented to the categories defined. We trained and evaluated our SFER model using the data collected from a human-in-the-loop simulation with a full-scale driving simulator. We proposed the DRER model by combining the best performing FER model model and SFER model in our experiments. As a result, our proposed DRER model achieved 88.6% accuracy using only the face image and EDA signal for the driver’s induced emotion while driving situation. Therefore, our DRER model is expected to be more reliable than the existing FER models and will be useful for services based on the driver’s emotional state.

There are multiple directions along which our proposed models could be robust in future work. The first is considering emotional continuity. Our DRER model recognized emotional state with only a short moment’s information, but the emotions have a continuous nature. Thus, if our proposed model considered the sequential aspect, its recognition performance would likely significantly improve to recognize driver’s emotion. Second, based on experiments using real vehicle, the robustness of the algorithm can be considerably improved with respect to the external environment. Even though the full-scale driving simulator that we used is very similar to the real vehicle environment, the external environment is completely controlled. It is likely that a future model trained with many data collected from the real vehicle can show high accuracy in uncontrolled actual driving situations. Third, in this study, we did not consider real-time recognition. Optimizing the frequency and time window through run time analysis would likely make it possible to build a real-time system for monitoring the real emotions of drivers.

## Figures and Tables

**Table 1:** Terminologies and definitions of the variable used in this paper.

Expression	Definition
ID	The variable to distinguish the individual driver
$I_{t}$	Driver’s face image at time *t* while driving
${\hat{V}}_{t}$	Driver’s recognized valence level by facial expressions at time *t*
${\hat{A}}_{t}$	Driver’s recognized arousal level by facial expressions at time *t*
$E_{t}$	Driver’s measured EDA response while driving at time *t*
*k*	Number of defined emotional state categories for driver
*C*	List of defined emotional state categories for driver, $C^{T}=\left[C_{1},C_{2},\ldots ,C_{k}\right]$
${\hat{Y}}_{t}$	Driver’s recognized real emotion while driving at time *t* among the *C*

**Table 2:** The configurations of trainable layers for the proposed FER model.

VGG14		VGG17		ResNet18		ResNet34		ResNet50		ResNet101		ResNeXt34		SE-ResNet34		SE-ResNeXt34	
3×3,64	×1	3×3,64	×2	7×7,64	×1	7×7,64	×1	7×7,64	×1	7×7,64	×1	7×7,64	×1	7×7,64	×1	7×7,64	×1
3×3,238	×2	3×3,128	×2	$\left.\begin{array}{c} 3\times 3,64\\ 3\times 3,64 \end{array}\right\}$	×2	$\left.\begin{array}{c} 3\times 3,64\\ 3\times 3,64 \end{array}\right\}$	×3	$\left.\begin{array}{c} 1\times 1,64\\ 3\times 3,64\\ 1\times 1,256 \end{array}\right\}$	×3	$\left.\begin{array}{c} 1\times 1,64\\ 3\times 3,64\\ 1\times 1,256 \end{array}\right\}$	×3	$\left.\begin{array}{c} 3\times 3,64\\ 3\times 3,2\times 32 \end{array}\right\}$	×3	$\left.\begin{array}{c} 3\times 3,64\\ 3\times 3,64\\ \mathrm{SE}\;\mathrm{block} \end{array}\right\}$	×3	$\left.\begin{array}{c} 3\times 3,64\\ 3\times 3,2\times 32\\ \mathrm{SE}\;\mathrm{block} \end{array}\right\}$	×3
3×3,256	×3	3×3,256	×4	$\left.\begin{array}{c} 3\times 3,128\\ 3\times 3,128 \end{array}\right\}$	×2	$\left.\begin{array}{c} 3\times 3,128\\ 3\times 3,128 \end{array}\right\}$	×4	$\left.\begin{array}{c} 1\times 1,128\\ 3\times 3,128\\ 1\times 1,512 \end{array}\right\}$	×4	$\left.\begin{array}{c} 1\times 1,128\\ 3\times 3,128\\ 1\times 1,512 \end{array}\right\}$	×4	$\left.\begin{array}{c} 3\times 3,128\\ 3\times 3,4\times 32 \end{array}\right\}$	×4	$\left.\begin{array}{c} 3\times 3,128\\ 3\times 3,128\\ \mathrm{SE}\;\mathrm{block} \end{array}\right\}$	×4	$\left.\begin{array}{c} 3\times 3,128\\ 3\times 3,4\times 32\\ \mathrm{SE}\;\mathrm{block} \end{array}\right\}$	×4
3×3,512	×3	3×3,512	×4	$\left.\begin{array}{c} 3\times 3,256\\ 3\times 3,256 \end{array}\right\}$	×2	$\left.\begin{array}{c} 3\times 3,256\\ 3\times 3,256 \end{array}\right\}$	×6	$\left.\begin{array}{c} 1\times 1,256\\ 3\times 3,256\\ 1\times 1,1024 \end{array}\right\}$	×6	$\left.\begin{array}{c} 1\times 1,256\\ 3\times 3,256\\ 1\times 1,1024 \end{array}\right\}$	×23	$\left.\begin{array}{c} 3\times 3,256\\ 3\times 3,8\times 32 \end{array}\right\}$	×6	$\left.\begin{array}{c} 3\times 3,256\\ 3\times 3,256\\ \mathrm{SE}\;\mathrm{block} \end{array}\right\}$	×6	$\left.\begin{array}{c} 3\times 3,256\\ 3\times 3,8\times 32\\ \mathrm{SE}\;\mathrm{block} \end{array}\right\}$	×6
3×3,512	×3	3×3,512	×4	$\left.\begin{array}{c} 3\times 3,512\\ 3\times 3,512 \end{array}\right\}$	×2	$\left.\begin{array}{c} 3\times 3,512\\ 3\times 3,512 \end{array}\right\}$	×3	$\left.\begin{array}{c} 1\times 1,512\\ 3\times 3,512\\ 1\times 1,2048 \end{array}\right\}$	×3	$\left.\begin{array}{c} 1\times 1,512\\ 3\times 3,512\\ 1\times 1,2048 \end{array}\right\}$	×3	$\left.\begin{array}{c} 3\times 3,512\\ 3\times 3,16\times 32 \end{array}\right\}$	×3	$\left.\begin{array}{c} 3\times 3,512\\ 3\times 3,512\\ \mathrm{SE}\;\mathrm{block} \end{array}\right\}$	×3	$\left.\begin{array}{c} 3\times 3,512\\ 3\times 3,16\times 32\\ \mathrm{SE}\;\mathrm{block} \end{array}\right\}$	×3
Fully connected with 2 units, tanh																	

Note: Two columns describe the information of each model: the left column shows the CNN filter and depth information and the right column shows the number of repetitions. The last row means the output layer that all have in common.

**Table 3:** Configurations of the proposed SFER models.

E(3, 64)	E(8, 512)	VA(3, 64)	VA(8, 512)	VAE(3, 64)	VAE(8, 512)	VAE(9, 1024)	VAE(9, 2048)	VAE(10, 1024)
32, ReLU	32, ReLU	32, ReLU	32, ReLU	32, ReLU	32, ReLU	32, ReLU	32, ReLU	32, ReLU
64, ReLU	64, ReLU	64, ReLU	64, ReLU	64, ReLU	64, ReLU	64, ReLU	128, ReLU	64, ReLU
32, ReLU	128, ReLU	32, ReLU	128, ReLU	32, ReLU	128, ReLU	128, ReLU	512, ReLU	128, ReLU
	256, ReLU		256, ReLU		256, ReLU	256, ReLU	1024, ReLU	512, ReLU
	512, ReLU		512, ReLU		512, ReLU	512, ReLU	2048, ReLU	512, ReLU
	256, ReLU		256, ReLU		256, ReLU	1024, ReLU	2048, ReLU	1024, ReLU
	64, ReLU		64, ReLU		64, ReLU	256, ReLU	256, ReLU	1024, ReLU
	32, ReLU		32, ReLU		32, ReLU	64, ReLU	128, ReLU	256, ReLU
						32, ReLU	32, ReLU	64, ReLU
								32, ReLU
8, softmax.								

Note: A column describe the information of each model and shows the number of units and activation function; the last row means the output layer that all have in common.

**Table 4:** RMSE values of valence and arousal on the validation set of the proposed FER.

Model		RMSE	
Base	Layer	Valence	Arousal
VGG	14	0.562	0.456
VGG	17	0.570	0.461
ResNet	34	0.418	0.378
ResNet	50	0.422	0.384
ResNet	101	0.420	0.389
ResNeXt	34	0.416	0.372
SE-ResNet	34	0.419	0.377
SE-ResNeXt	34	0.408	0.373

**Table 5:** The accuracy of the SFER model on test set.

Model	Accuracy
E(3,64)	0.331
E(8,512)	0.358
VA(3,64)	0.376
VA(8,512)	0.411
VAE(3,64)	0.658
VAE(8,512)	0.880
VAE(9,1024)	0.886
VAE(9,2048)	0.865
VAE(10,1024)	0.871

**Table 6:** Confusion matrix of the evaluate result of VAE(9,1024).

		*Y*							
		Neutral	Happy	Excited	Fearful	Angry	Depressed	Bored	Relieved
$\hat{Y}$	Neutral	0.877	0.006	0.013	0.011	0.016	0.017	0.015	0.031
	Happy	0.026	0.930	0.036	0.013	0.017	0.024	0.017	0.005
	Excited	0.011	0.012	0.873	0.019	0.017	0.026	0.013	0.003
	Fearful	0.015	0.007	0.016	0.883	0.031	0.025	0.013	0.020
	Angry	0.026	0.009	0.015	0.034	0.888	0.024	0.022	0.026
	Depressed	0.014	0.019	0.011	0.012	0.006	0.861	0.015	0.008
	Bored	0.013	0.012	0.025	0.013	0.013	0.017	0.883	0.014
	Relieved	0.018	0.004	0.012	0.015	0.012	0.006	0.022	0.892

Note: *Y* is the induced emotion categories to driver and $\hat{Y}$ is the recognized real emotion categories.

**Table 7:** AUC result of the proposed SFER models.

	VAE(9, 1024)	VAE(8, 512)	VA(8, 512)	E(8, 512)
Neutral	0.993	0.991	0.794	0.754
Happy	0.995	0.994	0.826	0.758
Excited	0.993	0.993	0.829	0.770
Fearful	0.992	0.992	0.813	0.818
Angry	0.991	0.990	0.845	0.756
Depressed	0.994	0.994	0.850	0.703
Bored	0.993	0.992	0.807	0.723
Relieved	0.995	0.994	0.817	0.822
Average AUC	0.993	0.993	0.823	0.764

**Table 8:** State-of-the-art algorithms for emotion recognition.

Reference	Signals	Number of Categories	Method	Accuracy
Machot et al. [22]	EDA	4 (Emotions)	CNN	0.81
Santamaria et al. [23]	ECG, EDA	2 (Valence)	DCNN	0.75
		2 (Arousal)		0.76
Rayatdoost et al. [24]	EEG, Faces	2 (Valence)	MGIF	0.75
		2 (Arousal)		0.74
Siddharth et al. [25]	EEG, Faces	4 (Emotions)	VGG-16, LSTM	0.54
Comas et al. [27]	EDA, Faces, ECG	7 (Emotions)	BMMN-BAE-2	0.64
Our study	EDA, Faces	8 (Emotions)	DRER	0.89

## Abbreviations

The following abbreviations are used in this manuscript:

DRER	Driver’s Real Emotion Recognizer
FER	Facial Expressions Recognition
SFER	Sensor Fusion Emotion Recognition
EEG	Electroencephalogram
ECG	Electrocardiogram
PPG	Photoplethysmography
EDA	Electrodermal activity
CNN	Convolutional neural network
DNN	Deep neural network
ROC	Receiver Operating Characteristic
AUC	Area Under the Curve
